# Predictors of Essential Health and Nutrition Service Delivery in Bihar, India: Results From Household and Frontline Worker Surveys

**DOI:** 10.9745/GHSP-D-14-00144

**Published:** 2015-06-12

**Authors:** Katrina Kosec, Rasmi Avula, Brian Holtemeyer, Parul Tyagi, Stephanie Hausladen, Purnima Menon

**Affiliations:** ^a^​ International Food Policy Research Institute, Washington, DC, USA; ^b^​ International Food Policy Research Institute, New Delhi, India; ^c^​ Harvard University Graduate School of Education, Cambridge, Massachusetts, USA

## Abstract

**Background:**

In Bihar, India, coverage of essential health and nutrition interventions is low. These interventions are provided by 2 national programs—the Integrated Child Development Services (ICDS) and Health/National Rural Health Mission (NRHM)—through Anganwadi workers (AWWs) and Accredited Social Health Activists (ASHAs), respectively. Little is known, however, about factors that predict effective service delivery by these frontline workers (FLWs) or receipt of services by households. This study examined the predictors of use of 4 services: (1) immunization information and services, (2) food supplements, (3) pregnancy care information, and (4) general nutrition information.

**Methods:**

Data are from a 2012 cross-sectional survey of 6,002 households in 400 randomly selected villages in 1 district of Bihar state, as well as an integrated survey of 377 AWWs and 382 ASHAs from the same villages. For each of the 4 service delivery outcomes, logistic regression models were specified using a combination of variables hypothesized to be supply- and demand-side drivers of service utilization.

**Results:**

About 35% of households reported receiving any of the 4 services. Monetary immunization incentives for AWWs (OR = 1.55, CI = 1.02–2.36) and above-median household head education (OR = 1.39, CI = 1.05–1.82) were statistically significant predictors of household receipt of immunization services. Higher household socioeconomic status was associated with significantly lower odds of receiving food supplements (OR = 0.87, CI = 0.79–0.96). ASHAs receiving incentives for institutional delivery (OR = 1.52, CI = 0.99–2.33) was marginally associated with higher odds of receiving pregnancy care information, and ASHAs who maintained records of pregnant women was significantly associated with households receiving such information (OR = 2.25, CI = 1.07–4.74). AWWs receiving immunization incentives was associated with significantly higher odds of households receiving general nutrition information (OR = 1.92, CI = 1.08–3.41), suggesting a large spillover effect of incentives from product- to information-oriented services.

**Conclusion:**

Product-oriented incentives affect delivery of both product- and information-oriented services, although household factors are also important. In India, existing government programs can mitigate supply- and demand-side constraints to receiving essential interventions by optimizing existing incentives for FLWs in national programs, helping FLWs better organize their work, and raising awareness among groups who are less likely to access services.

## Abstract (precis)

Only about 35% of sample households reported receiving immunization, food supplements, pregnancy care information, or nutrition information. Monetary incentives for such product-oriented services as immunization improved performance and may have spillover effects for information-oriented services. Immunization day events and good frontline worker recordkeeping also improved service delivery.

## INTRODUCTION

Globally, undernutrition is the underlying cause each year of some 3.1 million deaths in children under 5 years old (45% of all deaths in this group).^1^ Delivering 10 evidence-based essential interventions in such areas as adolescent and pregnancy nutrition, infant and young child feeding practices, immunization, and micronutrient supplementation at 90% coverage could reduce these total deaths by an estimated 15%.^1^

Community-based strategies are recognized as important for delivering such health and nutrition interventions—particularly those that require household behavioral changes.^2^ Specifically, frontline workers (FLWs)—paid or volunteer nonprofessional community health care providers^3^—can improve access to essential interventions^4^ and have been effective at preventing and managing neonatal childhood illnesses.^5^^,^^6^ Behavioral change interventions such as promotion of breastfeeding and hand washing,^4^ as well as instruction on the value of immunization,^7^ micronutrients, and insecticide-treated nets, can be delivered in the community by trained health professionals or by lay workers.^4^

Community-based strategies are important for delivering health interventions requiring household behavioral changes.

FLW roles are quite diverse; some FLWs provide a wide range of preventive and curative services while others provide only selected services.^8^ Careful selection and training, continued support including monetary and non-monetary incentives, establishing routine contact with families in the community, and monitoring are critical for a successful FLW program.^2^^,^^8^^–^^11^ Supportive supervision is also recognized to improve work productivity of FLWs providing preventive or curative services.^12^ There is, however, limited understanding of the differing determinants of effective FLW service delivery for preventive vs. curative services. Some researchers have noted that FLW workloads and preferences can play a role in shaping which services FLWs prioritize for delivery.^13^^–^^15^

In India, all essential health and nutrition interventions are delivered by FLWs through 2 national programs: the Integrated Child Development Services (ICDS) scheme and the Health/National Rural Health Mission (NRHM). Two types of FLWs are responsible for delivering services to communities: Anganwadi workers (AWWs) and Accredited Social Health Activists (ASHAs) (Table 1).

**TABLE 1.** Roles and Responsibilities of Frontline Workers in Bihar, India

**Anganwadi Workers (AWWs)**	**Accredited Social Health Activists (ASHAs)**
**Employment Terms**	
Part-time female workers recruited from local communities Employed by the Integrated Child Development Services Receive fixed monthly honorarium based on educational qualifications May receive monetary incentives for specific activities Encouraging families to use immunization Encouraging families to use family planning services Attending training sessions	Part-time female workers recruited from local communities Employed by the National Rural Health Mission Non-salaried but receive monetary incentives for specific activities Promoting universal immunization Promoting institutional deliveries Referring clients to RCH & other programs Constructing household toilets
**Education**	
Have at least 10th-grade education	Literate, preferably with at least 10th-grade education
**Catchment Area**	
Deliver services through Anganwadi centers, serving catchment area of 1,000 population	Deliver services in same catchment area as AWWs through home visits or in AWC
**Services Provided**	
Facilitate immunization Provide monthly food supplements to pregnant women, lactating mothers, and children Provide nutrition and health education, including information on pregnancy care and infant and child feeding practices, at AWCs and during home visits Plan and organize immunization days and VHNDs once a month with ASHAs Provide non-formal preschool education Facilitate health check-ups Provide referral services	Motivate families to use immunization Motivate pregnant women to have institutional deliveries Provide information on pregnancy, newborn, and infant care (including nutrition information) Provide other services related to communicable diseases Plan and organize immunization days and VHNDs once a month with AWWs Motivate families to use family planning

Abbreviations: AWCs, Anganwadi centers; RCH, reproductive and child health; VHNDs, Village Health and Nutrition Days.

AWWs are part-time female ICDS workers who receive a fixed monthly honorarium based on their educational qualifications and who may also receive monetary incentives for specific activities such as attending training sessions (payment per training attended) and encouraging families to use immunization and family planning services (payment per beneficiary vaccinated or referred, respectively). AWWs have at least a 10th-grade education and are recruited from local communities. They deliver services through Anganwadi centers (AWCs), serving a catchment area of a population of 1,000. AWWs provide monthly food supplements, facilitate immunization and health check-ups, and provide referral services, non-formal preschool education, and nutrition and health education (including information on pregnancy care and infant and child feeding practices)—both at the AWC and during home visits. Under the supplementary nutrition service, AWWs are responsible for providing ready-to-eat food supplements on the 15th day of every month to all pregnant and lactating mothers and children under 3 years old as well as hot cooked meals on at least 25 days each month to 3–6-year-old children at the AWCs. In Bihar state, this remains a more limited service where guidelines note that supplementary foods are to be provided at each AWC to only 28 malnourished children and to 12 severely and acutely malnourished children aged 6 months to 3 years, 16 pregnant and lactating women, and 3 adolescent girls.^16^

ASHAs are the non-salaried, part-time FLWs of the NRHM. They are literate women from the local communities, who are 25–45 years old and who preferably have at least a 10th-grade education. Once hired as ASHAs, they receive in-service training and attend monthly meetings. ASHAs deliver services in the same catchment areas (population of 1,000) as the AWWs, and receive performance-based incentives for promoting universal immunization, motivating pregnant women to have institutional deliveries, referring clients to Reproductive & Child Health (RCH) and other health care programs, and constructing household toilets.^17^ As with AWWs, the incentive amount ASHAs receive for providing a specific service is fixed and paid for each beneficiary who receives the service.^18^ ASHAs are expected to motivate families to use immunization, family planning, and institutional delivery; provide information on pregnancy, newborn, and infant care (including nutrition information; and provide other services pertaining to communicable diseases. Additionally, AWWs and ASHAs together plan and organize immunization days as well as Village Health and Nutrition Days (VHNDs) once a month, at which ICDS and health services are delivered.

Despite the systems in place for delivering essential health and nutrition services to communities, the poor state of service delivery for nutrition in India has been noted by several authors,^19^^,^^20^ and the problem is worse in states such as Bihar—one of India’s poorest. For example, only 28% of children under 6 months are exclusively breastfed, only a third of children are fully immunized, and only 23% of 6–23-month-old children are fed according to the World Health Organization’s recommendations. Furthermore, only 0.6% of pregnant and lactating women reported receiving food supplements, and only 0.2% reported receiving health and nutrition education.^21^

In this context, understanding factors that predict provision and receipt of services can provide direction to improve service delivery. We conducted a study in Bhojpur district of Bihar to examine the use of 4 services delivered through the FLWs of the ICDS and NRHM: (1) immunization services, (2) food supplements, (3) pregnancy care information, and (4) general nutrition information. In this article, we refer to immunization information and services and food supplements as **product-oriented services**, as they involve a product or the provision of information aimed solely at increasing uptake of a specific product. AWWs and ASHAs are expected to sensitize and motivate families to use immunization services; auxiliary nurse-midwives (ANMs) are responsible for providing the vaccines. Only AWWs provide food supplements, through AWCs. We consider pregnancy care and general nutrition information to be **information-oriented services**, as they require information provision and counseling without a specific product being associated with the information. AWWs and ASHAs are expected to provide this information at the AWCs and during home visits. Understanding the different factors influencing delivery of each service type can help identify strategies tailored to the specific features of a given service, to ultimately help improve its delivery.

Differentiating between product- and information-oriented services can help identify factors influencing delivery of each type of service.

## METHODS

### Hypotheses

In this analysis, we tested 3 main hypotheses concerning household receipt of specific health and nutrition services:

1. Providing FLWs with monetary incentives for service provision can increase delivery of those and other closely associated services
2. Greater health and nutrition information and knowledge on the part of FLWs will increase delivery of information-oriented services
3. Household characteristics predict receipt of food supplements (given the country’s goal for universal access to food supplementation programs but within a targeted approach that identifies the most needy, in the case of Bihar)

### Data Sources

The data for this study are from 2012 and come from household and FLW surveys conducted in 400 randomly selected villages located in 14 blocks of Bhojpur district in Bihar State. Similar trends are observed in Bhojpur as in the state of Bihar overall. About a third of the children in Bhojpur are fully immunized. The coverage for DPT3 is about 54% in Bihar, while it is 49% in Bhojpur.^22^

We added a brief module on the receipt of health and nutrition services during the last 3 months to an ongoing household survey that formed the baseline for a recent impact evaluation.^23^ The household survey covered 15 randomly sampled households in each village, for a total of 6,002 households. The survey gathered information on household composition, demographics, and food consumption, among other variables relevant to the main impact evaluation.

The survey of FLWs was conducted specifically for this study. We attempted to interview 1 AWW and 1 ASHA per survey village. If a village had more than 1 AWW or ASHA, we interviewed the worker serving the main village. If it was still unclear whom should be interviewed, we randomly chose one of the workers. For the few villages lacking a local AWW or ASHA, we used information on the AWW or ASHA most frequently serving the village. In total, we interviewed 377 of 394 AWWs and 382 of 396 ASHAs. Of the 17 AWWs not interviewed, 15 were not found and 2 were temporarily unavailable. Of the 14 ASHAs not interviewed, 10 were not found, 3 were temporarily unavailable, and 1 refused to participate. The AWW and ASHA surveys provide information on demographics, education, experience, knowledge, training, available equipment and resources, recordkeeping practices, monetary incentives, and the monitoring, support, and supervision they received.

We merged the household and FLW datasets to generate a household-level dataset containing information on both service users and their providers. We focused on outcomes for households with women and young children, thus restricting our analysis sample to between 500 and 2,038 observations, depending on the outcome.

### Dependent Variables

The dependent variables were constructed using questions regarding receipt of services from government service centers and at home. The analytic sample for each outcome was restricted to a subset of households, based on their eligibility to receive the services.

- **Outcome 1: Immunization services.** Households that reported receiving any immunization in the last 3 months at the AWC, sub-center, primary health center (PHC), or from an ANM at home. **Sample:** Only households with at least 1 child under 24 months of age were included (N = 1,199), because according to the national immunization schedule,^24^ most children in this age range should have received at least 1 immunization in the last 3 months.
- **Outcome 2: Food supplements.** Households that reported receiving food supplements at the AWC in the last 3 months. **Sample:** Households with at least 1 pregnant woman or with at least 1 child between 6 months and 3 years old (N = 2,038)
- **Outcome 3: Information on proper care during pregnancy.** Households that reported receiving pregnancy care information from an AWW, ASHA, or ANM at home in the last 3 months. **Sample:** Households with at least 1 pregnant woman or with at least 1 child 0–3 months old (N = 500)
- **Outcome 4: Information on general nutrition.** Households that reported receiving breastfeeding, child feeding, and general nutrition information at home from AWWs or ASHAs in the last 3 months. **Sample:** Households with at least 1 pregnant woman or with at least 1 child under 6 years old (N = 1,764)

As the focus of the study was to understand the determinants of *service delivery*, and the household data are from a secondary dataset, data from the dependent variables do not include standard indicators used to assess the *outcomes* of those services (for example, full immunization, actual pregnancy care, and child feeding practices). We also lacked data to construct such indicators.

### Independent Variables

For each of the 4 outcomes, we specified a different set of independent variables determined by their relevance to the delivery of the service. Some of the services are primarily the domain of either the AWW (food supplements) or ASHA (pregnancy care information), while others are provided by both (promoting immunization and providing general nutrition information). In addition, it is possible that different factors affect the delivery of product- vs. information-oriented services. For example, delivery of product-oriented services requires specific facilities and equipment (such as weighing scales to give food supplements). The provision of information-oriented services requires specific knowledge on the part of providers (for example, information on recommended breastfeeding practices).

The complete set of control variables comprised:

1. **Education and experience variables:** These include indicator variables for the worker having the median level of education or higher (class 10+ for ASHAs and class 11+ for AWWs) and the median number of years of experience or higher (6+ for ASHAs and 11+ for AWWs).
2. **Monetary environment variables:** All of these variables were constructed based on FLW self-reports of whether they had *ever* received incentives (for immunization, training, or institutional deliveries)—not whether they are eligible to receive them. They were not coded based on whether the FLW received incentives in conjunction with the services provided to any particular household surveyed. The guidelines on payments for ASHAs in Bihar are unclear. Payments for institutional deliveries by *Janani Suraksha Yojana* (JSY) (the NRHM safe motherhood intervention) are made in cash, while payments for other services are made by check or wire transfer.^25^ Only for institutional delivery incentives did we know the actual amount received. Furthermore, payment of incentives is not uniform across villages for several reasons. First, receipt of incentives requires knowledge on the part of FLWs about the payments to which they are entitled. An effective system to record and monitor what services FLWs have delivered is also needed, in addition to actually delivering the incentive payments. A breakdown in this process can lead to a failure to receive incentives for work performed. Furthermore, incentives for AWWs in Bihar were meant to be gradually eliminated around 2011–2012, as ASHAs became more firmly entrenched as the FLWs receiving performance-based incentives. Some blocks, and some villages within blocks, appear to have phased out incentives for AWWs more quickly than others for financial and logistical reasons, generating variation in access to incentives by AWWs.
3. **Effort and organization variables:** These include indicator variables for holding designated immunization days and VHNDs, keeping immunization and pregnancy registries, attending monthly FLW meetings, and having a weighing device for food.
4. **Supervision, training, and knowledge variables:** These include indicator variables for FLWs correctly identifying the position their supervisor occupies, receiving training on pregnancy- and nutrition-related topics in either their first or last training, listing pregnancy as one use of iron pills, and knowing the proper ages to start liquids other than breast milk and solid foods.
5. **Household and village control variables:** These include household head education, household socioeconomic status quintile, variables recording whether the caste of the household head is the same as that of the ASHA and of the AWW, number of pregnant women in the catchment area (10s), and village population (1,000s).

### Analysis

Logistic regression models were specified based on theoretical and programmatic considerations for each of the 4 service delivery outcomes, using a combination of variables:

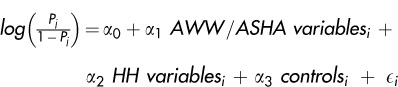

Where *P_j_* is the probability that household *i* accesses service *j* and controls included the village population and dummies for the season and block of residence. In all models, standard errors were clustered at the village level, as the availability and quality of health and nutrition services vary at this level and thus influence all household decisions within the village. This corrects for heteroskedasticity and allows for correlation of errors within villages.

## RESULTS

### Characteristics of Frontline Workers

Table 2 summarizes the key independent variables across the 377 AWWs and 382 ASHAs for which we have data. AWWs were more experienced than ASHAs; just over half of ASHAs had 6 or more years of experience, while just over half of AWWs had 11 or more years of experience. With regard to the incentive environment, only about 25% of AWWs reported ever receiving incentives for immunizations and 37% reported ever receiving training incentives. That such incentives were received by only selected AWWs is due both to imperfect policy implementation and a slow and non-uniform effort in phasing out AWW monetary incentives, taking place at the time of our survey. About 88% of AWWs held a designated immunization day, and 59% of ASHAs had a registry of pregnant women in the village.

About one-quarter of AWWs reported ever receiving immunization incentives.

**TABLE 2.** Characteristics of ASHAs and AWWs in Bhojpur District, Bihar State, India

	**Value**	**N**
**Education and experience**		
ASHAs who completed class 10+, %	43.5	382
AWWs who completed class 11+, %	39.3	377
ASHAs who have 6+ years of experience, %	52.6	382
AWWs who have 11+ years of experience, %	54.1	377
**Monetary incentives**		
AWWs who have ever received immunization incentives, %	24.9	370
AWWs who have ever received training incentives, %	37.1	356
Amount ASHAs receive for institutional delivery (100s), mean, rupees	3.91	369
AWWs who have experienced frequent delays in payments for immunizations, %	46.7	360
AWWs who have experience delayed payment for food supplements sometime in last 6 months, %	64.6	370
Primary reason for working is income generation, ASHAs, %	78.3	374
Primary reason for working is income generation, AWWs, %	61.3	372
**Worker effort and organization**		
AWWs who have held a designated immunization day, %	87.5	377
AWWs who have held a Village Health and Nutrition Day, %	7.4	376
ASHAs who keep children's immunization registry, %	88.2	382
AWWs who keep children's immunization registry, %	97.6	370
ASHAs who keep registry of pregnant women in village, %	58.9	382
ASHAs who attended 6+ meetings in past 6 months, %	74.3	382
AWWs who have weighing device for food, %	89.7	377
**Supervision, training, and knowledge**		
ASHAs who know who their supervisor is, %	45.1	381
AWWs who know who their supervisor is, %	92.6	377
ASHAs who have received training on pregnancy-related topics, %	70.5	308
ASHAs who have received training on nutrition-related topics, %	45.7	302
AWWs who have received training on nutrition-related topics, %	71.0	365
ASHAs who list pregnancy as one use of iron pills, %	84.6	382
ASHAs who know age to start liquids other than breast milk, %	86.6	382
AWWs who know age to start liquids other than breast milk, %	88.0	376
ASHAs who know age to start food other than breast milk, %	50.0	382
AWWs who know age to start food other than breast milk, %	66.0	377
**Household and village variables**		
Pregnant women in catchment area (10s), mean	1.52	374
Village population (1,000s), mean	2.07	354

Abbreviations: ASHAs, Accredited Social Health Activists; AWWs, Anganwadi workers.

### Receipt of Product- and Information-Oriented Services

More households reported receiving product-oriented services than information-oriented services. In the sample used to assess outcome 1 (immunization services), 31% of households reported receiving immunization in the last 3 months, and of those, about 46% reported receiving them at the AWC (Table 3). About 13% of the outcome 2 (food supplements) sample reported receiving food supplements. In contrast, only 11% of the outcome 3 sample reported receiving information on proper care during pregnancy, and only 5% of the outcome 4 sample reported receiving general nutrition information (Table 3). Looking over all households in any one of the four samples—that is, looking at households with either a pregnant woman or a child under age 6—about 35% reported receiving any of the 4 services, either at home or at a government facility.

About 35% of households reported receiving any of the 4 health services.

**TABLE 3.** Percentage of Households Reporting Receipt of Health and Nutrition Services, Bhojpur District, Bihar State, India

	**Service and Surveyed Sample**			
	**Immunization**	**Food Supplements**	**Pregnancy Care Information**	**General Nutrition Information**
	**HHs with children 0–2 y (N = 1,199)**	**HHs with pregnant women or children 6 m–3 y (N = 2,038)**	**HHs with pregnant women or children 0–3 m (N = 500)**	**HHs with pregnant women or children under 6 y (N = 1,764)**
Received service	31	13	11	5
Received service at government facilities				
AWCs	46	100	NA	NA
PHCs	7	NA	NA	NA
SCs	8	NA	NA	NA
Received service during home visits via:				
ANMs	48	NA	21	NA
ASHAs	NA	NA	72	47
AWWs	NA	NA	15	78

Abbreviations: ANMs, auxiliary nurse-midwives; ASHAs, Accredited Social Health Activists; AWCs, Anganwadi centers; AWWs, Anganwadi workers; HHs, households; NA, not applicable (service not available at that location or through that provider cadre); PHC, primary health center; SCs, sub-centers.

### Correlates of Using Product-Oriented Services

#### Immunization Services

Among households in the outcome 1 (immunization services) sample, just under a quarter had AWWs who had received immunization incentives, and 38% had AWWs who had received training incentives (Table 4). About half (47%) resided in villages where AWWs reported frequent delays in payments for immunizations. Almost 90% of such households lived in villages where the AWW had held a designated immunization day, while only 8% lived in villages where the AWW had held a VHND.

**TABLE 4.** Relationship Between Frontline Worker and Household Characteristics and Household Receipt of Health and Nutrition Services, Bhojpur District, Bihar State, India

	**Service and Sample Reporting Receipt of Service**			
	**Immunization**	**Food Supplements**	**Pregnancy Care Information**	**General Nutrition Information**
****	**HHs with children 0–2 y (N = 1,199)**	**HHs with pregnant women or children 6 m–3 y (N = 2,038)**	**HHs with pregnant women or children 0–3 m (N = 500)**	**HHs with pregnant women or children under 6 y (N = 1,764)**
**Education and experience, % (95% CI)**				
ASHAs who completed class 10+	46 (43–49)	NA	43 (39–47)	45 (42–47)
AWWs who completed class 11+	40 (37–43)	40 (38–42)	NA	40 (38–42)
ASHAs who have 6+ years of experience	56 (53–59)		66 (62–70)	65 (63–67)
AWWs who have 11+ years of experience	56 (53–58)	59 (57–61)	NA	59 (57–61)
**Monetary incentives, % (95% CI)**				
AWWs who have ever received immunization incentives	22 (20–25)	25 (23–26)	NA	22 (20–24)
AWWs who have ever received training incentives	38 (35–41)	40 (37–42)	NA	44 (42–46)
Amount ASHAs receive for institutional delivery (100s), rupees	NA	NA	3.95 (0.99)	3.88 (0.95)
AWWs who have experienced frequent delays in payments for immunizations	47 (44–50)	NA	NA	NA
AWWs who have experienced delayed payment for food supplements sometime in last 6 months	NA	65 (63–67)	NA	NA
Primary reason for working is income generation, ASHAs	79 (76–81)	NA	76 (73–80)	76 (74–78)
Primary reason for working is income generation, AWWs	60 (58–63)	62 (60–64)	NA	63 (60–65)
**Worker effort and organization, % (95% CI)**				
AWWs who have held a designated immunization day	89 (88–91)	NA	NA	NA
AWWs who have held a VHND	8 (7–10)	8 (7–9)	6 (4–8)	7 (6–8)
ASHAs who keep children's immunization registry	90 (88–91)	NA	NA	NA
AWWs who keep children's immunization registry	98 (97–99)	NA	NA	NA
ASHAs who keep registry of pregnant women in village	NA	NA	63 (59–68)	NA
ASHAs who attended 6+ meetings in past 6 months	79 (77–82)	NA	83 (79–86)	80 (78–82)
AWWs who have weighing device for food	NA	90 (89–91)	NA	NA
**Supervision, training, and knowledge, % (95% CI)**				
ASHAs who know who their supervisor is	46 (43–49)	NA	50 (46–55)	52 (50–55)
AWWs who know who their supervisor is	92 (91–94)	93 (92–94)		92 (91–94)
ASHAs who have received training on pregnancy-related topics	NA	NA	72 (68–76)	NA
ASHAs who have received training on nutrition-related topics	NA	NA	NA	46 (43–48)
AWWs who have received training on nutrition-related topics	NA	NA	NA	73 (71–75)
ASHAs who list pregnancy as one use of iron pills	NA	NA	83 (80–86)	NA
ASHAs who know age to start liquids other than breast milk				89 (88–91)
AWWs who know age to start liquids other than breast milk	NA	NA	NA	87 (86–89)
ASHAs who know age to start food other than breast milk	NA	NA	NA	49 (47–51)
AWWs who know age to start food other than breast milk	NA	NA	NA	69 (67–71)
**Household and village variables**				
ASHAs and household head of same caste, % (95% CI)	5.0 (3.8–6.2)	NA	5.0 (3.1–6.9)	5.8 (4.7–6.9)
AWWs and household head of same caste, % (95% CI)	5.1 (3.8–6.3)	5.9 (4.9–6.9)	NA	7.2 (6.0–8.4)
Pregnant women in catchment area (10s), mean (SD)	NA	NA	1.55 (0.80)	NA
Household heads who have completed class 7+, % (95% CI)	42 (39–45)	42 (40–44)	43 (38–47)	43 (41–45)
Household socioeconomic status index, mean (SD)	3.20 (1.41)	3.13 (1.44)	3.26 (1.42)	3.06 (1.43)
Village population (1,000s), mean (SD)	2.08 (2.34)	2.11 (2.38)	2.37 (2.75)	2.27 (2.58)

Abbreviations: ASHAs, Accredited Social Health Activists; AWWs, Anganwadi workers; NA, not applicable; SD, standard deviation; VHND, Village Health and Nutrition Day.

Prevalence rates and 95% confidence interval are reported for all binary variables. For non-binary variables, means and standard deviations are reported.

In multivariate logistic regression analysis of factors predicting household receipt of immunization services in the last 3 months, we found that households that were in villages where the AWW received monetary incentives for providing immunizations had a 55% higher chance of receiving immunization services than did households in villages without such incentives (confidence interval [CI] = 1.02–2.36; *P* = .04) (Table 5). Holding a designated immunization or VHND was not associated with receipt of immunization services, nor was greater educational attainment or years of experience of the workers.

Monetary incentives for immunization were significantly associated with household receipt of immunization services.

**TABLE 5.** Multivariate Logistic Regression Model Showing Predictors of Receipt of Immunization Services For Households With Children 0–2 Years, Bhojpur District, Bihar State, India (N = 1,199)

	**OR**	**95% CI**	***P***
**Education and experience**			
ASHAs who completed class 10+	0.929	0.694–1.244	.62
AWWs who completed class 11+	1.040	0.769–1.408	.80
ASHAs who have 6+ years of experience	0.853	0.636–1.144	.29
AWWs who have 11+ years of experience	1.120	0.786–1.595	.53
**Monetary incentives**			
*AWWs who have ever received immunization incentives*	*1.552*	*1.019–2.363*	.*04*
AWWs who have ever received training incentives	0.967	0.719–1.301	.83
AWWs who have experienced frequent delays in payments for immunizations	0.999	0.743–1.344	1.00
Primary reason for working is income generation, ASHAs	0.766	0.538–1.091	.14
Primary reason for working is income generation, AWWs	1.266	0.936–1.711	.13
**Worker effort and organization**			
AWWs who have held a designated immunization day	1.119	0.764–1.641	.56
AWWs who have held a VHND	1.078	0.695–1.670	.74
ASHAs who keep children's immunization registry	0.860	0.538–1.373	.53
AWWs who keep children's immunization registry	0.742	0.329–1.673	.47
ASHAs who attended 6+ meetings in past 6 months	0.936	0.663–1.322	.71
**Supervision, training, and knowledge**			
ASHAs who know who their supervisor is	0.999	0.746–1.338	1.00
AWWs who know who their supervisor is	0.906	0.501–1.640	.75
**Household and village variables**			
*ASHAs and household head of same caste*	*1.863*	*1.048–3.312*	.*03*
*AWWs and household head of same caste*	*0.445*	*0.217–0.913*	.*03*
*Household heads who have completed class 7+*	*1.385*	*1.052–1.823*	.*02*
Household socioeconomic status index	1.043	0.944–1.153	.41
*Village population (1,000s)*	*0.885*	*0.816–0.959*	*<.001*

Abbreviations: ASHAs, Accredited Social Health Activists; AWWs, Anganwadi workers; CI, confidence interval; OR, odds ratio; VHND, Village Health and Nutrition Day.

Variables shown in italics are statistically significant at *P* < .10.

Among household and village characteristics, an important predictor of household receipt of immunization services was the education level of the head of the household. Specifically, households with a head who had completed class 7 or higher had a 39% higher chance of receiving immunization services than did households with a less educated household head (CI = 1.05–1.82; *P* = .02) (Table 5). After adjusting for education, household socioeconomic status did not have a significant impact on receipt of immunization services (OR = 1.04, CI = 0.94–1.15; *P* = .41). The results on whether caste predicted receipt of immunizations were not consistent; when ASHAs and household heads were of the same caste, there was greater likelihood of receiving immunization services (OR = 1.86, CI = 1.05–3.31; *P* = .03), but when AWWs and household heads were of the same caste, there was lower likelihood of receiving immunization services (OR = 0.45, CI = 0.22–0.91; *P = .03*). Finally, a 1,000 person increase in the village population was associated with significantly lower use of immunization services (OR = 0.89, CI = 0.82–0.96; *P*<.01).

#### Food Supplements

Among households in the outcome 2 (food supplements) sample, almost two-thirds had AWWs who reported receiving a delayed payment for food supplements sometime in the last 6 months. However, 90% of households’ AWWs reported having a device for weighing food, and 93% knew their supervisor (Table 4).

In multivariate logistic regression analysis, there was no significant association between households receiving food supplements and any AWW characteristic (e.g., education, experience, knowing their supervisor), contextual factor (e.g., monetary incentives for immunization or training, on-time payment, holding VHNDs, having a food-weighing device), or most household or village characteristics (e.g., caste or education level of household head, or village size) (Table 6). The only factor that was significantly associated with household receipt of food supplements was household socioeconomic status: a 1-quintile increase in the household’s socioeconomic status index was associated with a 13% lower chance of the household receiving food supplements (OR = 0.87, CI = 0.79–0.96; *P* = .01).

**TABLE 6.** Multivariate Logistic Regression Model Showing Predictors of Receipt of Food Supplements For Households With Pregnant Women or Children 6 Months to 3 Years, Bhojpur District, Bihar State, India (N = 2,038)

	**OR**	**95% CI**	***P***
**Education and experience**			
AWWs who have completed class 11+	1.303	0.931–1.824	.12
AWWs who have 11+ years of experience	0.838	0.581–1.210	.35
**Monetary incentives**			
AWWs who have ever received immunization incentives	1.220	0.773–1.925	.39
AWWs who have ever received training incentives	0.885	0.633–1.237	.47
AWWs who have experience delayed payment for food supplements sometime in last 6 months	1.022	0.694–1.504	.91
Primary reason for working is income generation, AWWs	1.079	0.781–1.492	.64
**Worker effort and organization**			
AWWs who have held a VHND	0.847	0.497–1.446	.54
AWWs who have weighing device for food	1.275	0.735–2.211	.39
**Supervision, training, and knowledge**			
AWW knows who is their supervisor	1.599	0.812–3.151	.18
**Household and village variables**			
AWWs and household head of same caste	1.011	0.536–1.907	.97
Household heads who have completed class 7+	0.839	0.633–1.112	.22
*Household socioeconomic status index*	*0.871*	*0.789–0.962*	.*007*
Village population (1,000s)	1.015	0.972–1.060	.51

Abbreviations: AWWs, Anganwadi workers; CI, confidence interval; OR, odds ratio; VHND, Village Health and Nutrition Day.

Variables shown in italics are statistically significant at *P* < .10.

### Correlates of Using Information-Oriented Services

#### Information on Proper Care During Pregnancy

Among households in the outcome 3 (pregnancy care information) sample, 72% had ASHAs who had received training on pregnancy-related topics, and 83% had ASHAs who correctly listed pregnancy as one possible use of iron pills (Table 4). Further, 63% of such households had ASHAs who kept a registry of pregnant women. These ASHAs were paid almost 400 rupees (approximately US$7.50 in 2012), on average, for each institutional delivery under the JSY conditional cash transfer scheme.

Monetary incentives for institutional deliveries were associated with household receipt of pregnancy care information.

In multivariate logistic regression analysis, neither educational attainment nor years of experience of the ASHAs was significantly associated with receipt of pregnancy care information by the target population (Table 7). However, monetary incentives for institutional deliveries was significant: a 100-rupee increase in payment per delivery was associated with a 52% greater chance of households receiving pregnancy care information in the past 3 months (CI = 0.99–2.33; *P* = .06). There was no evidence that ASHAs being primarily motivated by income generation affected households’ receipt of pregnancy care information.

**TABLE 7.** Multivariate Logistic Regression Model Showing Predictors of Receipt of Pregnancy Care Information For Households With Pregnant Women or Children Under 3 Months, Bhojpur District, Bihar State, India (N = 500)

****	**OR**	**95% CI**	*****P*****
**Education and experience**			
ASHAs who have completed class 10+	1.113	0.591–2.094	.74
ASHAs who have 6+ years of experience	0.740	0.371–1.473	.39
**Monetary incentives**			
*Amount ASHAs receive for institutional delivery (100s)*	*1.519*	*0.989–2.334*	.*06*
Primary reason for working is income generation, ASHAs	1.095	0.535–2.241	.80
**Worker effort and organization**			
AWWs who have held a VHND	1.097	0.344–3.497	.88
*ASHAs who keep registry of pregnant women in village*	*2.254*	*1.072–4.740*	.*03*
ASHAs who have attended 6+ meetings in past 6 months	1.355	0.490–3.748	.56
**Supervision, training, and knowledge**			
ASHAs who know who their supervisor is	1.501	0.761–2.964	.24
ASHAs who have received training on pregnancy-related topics	0.580	0.249–1.355	.21
ASHAs who list pregnancy as one use of iron pills	1.180	0.482–2.888	.72
**Household and village variables**			
ASHAs and household head of same caste	1.198	0.392–3.659	.75
*Pregnant women in catchment area (10s)*	*0.495*	*0.260–0.942*	.*03*
Household heads who have completed class 7+	1.099	0.608–1.984	.76
Household socioeconomic status index	1.002	0.790–1.271	.99
Village population (1,000s)	1.076	0.985–1.175	.11

Abbreviations: ASHAs, Accredited Social Health Activists; AWWs, Anganwadi workers; CI, confidence interval; OR, odds ratio; VHND, Village Health and Nutrition Day.

Variables shown in italics are statistically significant at *P* < .10.

Households living in villages where ASHAs kept a register of pregnant women were more than twice as likely to receive pregnancy care information as were households in villages lacking such a register (OR = 2.25, CI = 1.07–4.74; *P* = .03). There was no significant association between receipt of pregnancy care information and any other worker effort, training, knowledge, or supervision factor.

Similarly, household and village characteristics were not important predictors of households receiving pregnancy care information except for the number of pregnant women in the catchment area. For every 10 additional pregnancies, households had 50% less chance of receiving pregnancy care information (CI = 0.26–0.94; *P* = .03).

#### General Nutrition Information

Among households in the outcome 4 (general nutrition information) sample, only 46% had ASHAs who had received training on nutrition-related topics, while 73% had AWWs who had received such training (Table 4). Similarly, only 49% of such households had ASHAs who knew the appropriate age to start foods other than breast milk, while 69% had AWWs who knew the appropriate age.

In multivariate logistic regression analysis, more years of work experience for AWWs (but not for ASHAs) was marginally associated with greater receipt of nutrition information by households: target households with an AWW having at least 11 years of experience had a 59% higher likelihood of receiving nutrition information than did target households with less experienced AWWs (CI = 0.92–2.73; *P* = .10) (Table 8). There was no significant association between receipt of general nutrition information and worker knowledge. However, receipt of monetary incentives by AWWs for providing immunizations was significantly associated with households receiving general nutrition information (OR = 1.92, CI = 1.08–3.41; *P* = .03).

Monetary incentives for immunization were significantly associated with household receipt of general nutrition information.

**TABLE 8.** Multivariate Logistic Regression Model Showing Predictors of Receipt of General Nutrition Information For Households With Pregnant Women or Children Under 6 Years, Bhojpur District, Bihar State, India (N = 1,764)

	**OR**	**95% CI**	***P***
**Education and experience**			
ASHAs who have completed class 10+	0.770	0.466–1.271	.31
AWWs who have completed class 11+	1.181	0.719–1.940	.51
ASHAs who have 6+ years of experience	0.764	0.456–1.279	.31
*AWWs who have 11+ years of experience*	*1.588*	*0.923–2.732*	.*10*
**Monetary incentives**			
*AWWs who have ever received immunization incentives*	*1.919*	*1.081–3.406*	.*03*
AWWs who have ever received training incentives	1.340	0.820–2.190	.24
Amount ASHAs receive for institutional delivery (100s)	0.988	0.748–1.303	.93
Primary reason for working is income generation, ASHAs	1.180	0.674–2.066	.56
Primary reason for working is income generation, AWWs	1.139	0.684–1.895	.62
**Worker effort and organization**			
AWWs who have held a VHND	1.182	0.432–3.239	.75
ASHAs who have attended 6+ meetings in past 6 months	0.903	0.520–1.571	.72
**Supervision, training, and knowledge**			
ASHAs who know who their supervisor is	1.179	0.698–1.992	.54
AWWs who know who their supervisor is	0.828	0.324–2.113	.69
ASHAs who have received training on nutrition-related topics	0.982	0.602–1.603	.94
AWWs who have received training on nutrition-related topics	1.300	0.769–2.197	.33
ASHAs who know age to start liquids other than breast milk	1.611	0.586–4.431	.36
AWWs who know age to start liquids other than breast milk	1.081	0.524–2.229	.83
ASHAs who know age to start food other than breast milk	1.093	0.677–1.765	.72
AWWs who know age to start food other than breast milk	0.792	0.487–1.289	.35
**Household and village variables**			
ASHAs and household head of same caste	1.477	0.524–4.162	.46
AWWs and household head of same caste	0.672	0.229–1.972	.47
Household heads who have completed class 7+	1.286	0.782–2.115	.32
Household socioeconomic status index	1.069	0.912–1.254	.41
Village population (1,000s)	0.957	0.865–1.058	.39

Abbreviations: ASHAs, Accredited Social Health Activists; AWWs, Anganwadi workers; CI, confidence interval; OR, odds ratio; VHND, Village Health and Nutrition Day.

Variables shown in italics are statistically significant at *P* < .10.

## DISCUSSION

This article describes one of the few studies to differentiate maternal and child health preventive services as *product-oriented* and *information-oriented* services. It examines both supply- and demand-side factors influencing the provision of each type of service using an integrated household and FLW dataset. Overall, results show that receipt of essential health and nutrition services remains low in Bihar, India, with only about 35% of study households (those with a pregnant woman or a child under age 6) reporting receiving any of 4 services: immunization, food supplements, information on proper care during pregnancy, or nutrition information. Although the outcomes in our study are not based on coverage indicators, the receipt of services are in congruence with the coverage indicators reported in other surveys.^21^ Receipt of information-oriented services was particularly low even though government program guidelines stipulate that FLWs are expected to conduct regular home visits to promote nutrition and health education. These findings are supported by other studies.^26^^–^^28^

### Monetary Incentives

In terms of our study hypotheses, first, we found that monetary incentives provided to FLWs for product-oriented services were associated with increased receipt of those services as well as a modest effect on receipt of information-oriented services that could be delivered during the same visit. For example, receipt of monetary incentives by FLWs for immunization was associated with households receiving immunization services as well as information on general nutrition. In addition, receipt of monetary incentives for institutional deliveries was marginally associated with households receiving information on proper care during pregnancy. This suggests possible spillover effects of incentives.

Monetary incentives for product-oriented services improved performance for that service and may also have had spillover effects for information-oriented services.

Given the poverty of such FLWs as AWWs and ASHAs,^29^ incentives may motivate them,^30^ reduce attrition,^8^^,^^10^ and improve performance.^4^ It is also plausible that when FLWs receive incentives to increase immunization and other product-oriented services, they take advantage of the opportunity to provide nutrition and other health information while in contact with these individuals. It is also plausible that incentives motivate FLWs to provide high-quality services and improve their outreach, which more generally raises households’ familiarity with FLWs and increases their likelihood of using services—including non-incentivized ones. More research is needed to understand what combinations of FLW incentives and policy or institutional reforms will best strengthen the delivery of health and nutrition services in Bihar, but incentives are likely to play a role.

Given such spillovers from product- to information-oriented services, our findings have especially important implications for cost-effectiveness analysis of performance-based incentives. In the context of overall low coverage of information-oriented services, the spillover effects are modest. Even a large percentage increase in the provision of information on proper care during pregnancy (with a base rate of access of 11%) or on general nutrition (with a base rate of access of 5%) translates to only a small number of additional individuals receiving such services. However, if India continues to provide monetary incentives for product-oriented services, our findings suggest that a large number of people would likely start to gain access to information-oriented services not specifically targeted by the incentives. As immunization incentives are already part of the existing incentive structure within the national program, policy should take into account how they might be best leveraged to improve the delivery of multiple services. Our findings contribute to the ongoing debate on the nature of incentives (i.e., cash, in-kind, or a combination) that are optimal for FLWs.^11^

It is important to note that while incentives for product-oriented services were associated with greater use of some information-oriented services, they were not associated with greater use of other product-oriented services. Future research is needed to understand what factors or service characteristics seem to maximize spillover impacts, which is relevant for designing more cost-effective incentive schemes that fully take into account both direct and spillover impacts of those schemes.

### Frontline Worker Knowledge and Delivery of Information Services

We did not find support for our second hypothesis, that greater FLW knowledge increases delivery of information-oriented services. However, the level of effort or organization of a FLW was an important predictor of households receiving pregnancy care information. Specifically, households living in villages where ASHAs kept a register of pregnant women were more than twice as likely to receive pregnancy care information as were households in villages lacking such a register. This may suggest that FLW knowledge is already at acceptable levels and that the effort and ability of FLWs are the more binding constraints on service delivery. Alternatively, the findings might suggest that given other constraints on service delivery, information and knowledge gaps of FLWs are not as important.

### Household Socioeconomic Status and Receipt of Food Supplements

With regards to our third hypothesis, household socioeconomic status did impact receipt of food supplements: households with lower socioeconomic status were more likely to receive food supplements, suggesting that food supplements are reaching the segment of population that needs them the most. This is contrary to the intent of the ICDS program of universalizing access to food supplements, but it is in line with the Bihar government’s guidelines for a targeted approach.^16^ It is important to note that the government’s guidelines are unclear on what form this targeted approach should take and exactly how malnourished targeted children should be. Because our study lacked data on child anthropometrics, we can neither confirm that the government has achieved its vision of how targeting should work, nor that child anthropometrics are improving in response to the employed targeting scheme.

Lower-income households were more likely to receive food supplements than were higher-income households, suggesting food supplements were reaching those most in need.

Receipt of food supplements was poor overall, which has been highlighted in other studies in Bihar as well. Such studies report the proportion of beneficiaries receiving supplements to be as low as 0.6%,^21^ and the total number of days beneficiaries receive supplements to be fewer than program guidelines require.^31^ The poor reach of food supplements is likely due to inadequate supplies,^29^ poor food quality, or lack of perceived need for additional food. Indeed, at the time of our study, the state of Bihar capped the resources to each AWW to provide food supplements to a fixed number of beneficiaries,^32^ likely leading to an ad hoc process of identifying the most needy beneficiaries. Given that only household socioeconomic status predicted receipt of food supplements, our results suggest that changes in the AWW’s operating environment itself may not matter much for whether households receive this product-oriented service.

### Predictors of Information-Oriented Services

In terms of information-oriented services, FLW experience and level of organization (i.e., keeping records), as well as the number of potential beneficiaries in the catchment area, were important predictors of receiving such services. ASHAs who maintained a registry for pregnant women were more likely to provide pregnancy care information. The need for adequate beneficiary registers to be able to provide necessary services has been recognized previously.^2^ In this context, maintaining a pregnancy registry could be indicative of an ASHA’s level of engagement with the community as well as motivation to track pregnant women to avail JSY incentives at the time of delivery. The higher the number of pregnant women in the catchment area, however, the less likely it was that pregnant women received information. This may indicate that FLWs’ resources and capacity to provide pregnancy information is strained by high demand for services. Areas with higher fertility rates may require additional human resources to ensure that pregnant women get appropriate information and counseling on their pregnancies.

### Predictors of Product-Oriented Services

In general, AWW and ASHA education, training, supervision, and experience were not significant predictors of receipt of product-oriented services, according with existing findings that preservice training is not an important determinant of the quality of care provided by FLWs.^33^ This finding may be good news because recruiting more educated and well-trained workers to deliver services could be expensive or not feasible. It merits noting, however, that existing literature identifies training and supervision as important factors for successful service delivery,^2^ high-quality care and communication by FLWs,^34^ and effective FLW performance more broadly.^35^ It is possible that we did not find similar effects due to contextual differences unique to India. Training content and its duration, rigor, and implications for service provision are determined by the larger context within which the program operates.^10^ Poor training, staff vacancies, and irregular supervision have been long-standing issues in India’s nutrition program,^19^ which may explain the insignificant effects in our study.

In terms of household characteristics or demand-side factors, households with more educated household heads were more likely to receive immunization services than those with lower educated household heads, possibly because educated household heads might be more aware of services and knowledgeable of the benefits of using the services. Other studies have found a positive association between maternal education and full immunization,^36^^,^^37^ according well with our results. Further, well-educated household heads might have the agency to access the services. One implication of our findings is that in the short-term, less educated households might need to be specifically targeted by outreach activities to enhance awareness of services such as immunization.

Our study showed mixed results on the role of caste in receipt of services. Caste match between ASHAs and the household head was a positive predictor of receipt of immunization services, but caste match between AWWs and the household head was a negative predictor of the same service. Families that are of similar caste as AWWs may belong to a higher social group and thus do not avail services from the AWCs or other government facilities, instead choosing to use private providers. On the other hand, ASHAs were introduced into their communities only after 2006 and hence are relatively new compared with AWWs who have been working in the same communities for much longer. ASHAs may prioritize working with families of their caste, since it might be easier for them to approach and convince such families to avail immunization services than families from other castes, given their relatively new role as FLWs in the community. Although our discrepant findings require further inquiry, there is evidence supporting the overall influence of social barriers such as caste, disability, and migrant status on service utilization.^27^ Addressing any systemic exclusions of particular groups and building trust requires long-range strategies that incorporate an awareness of any such exclusions.

## CONCLUSION

Existing government programs in India have the potential to increase use of essential health and nutrition interventions, but greater efforts are needed to mitigate supply- and demand-side constraints. While our study is narrow in geographic scope, it shows that incentivizing FLWs and helping them organize their work is associated with greater receipt of services by households, suggesting that effective implementation of existing program elements has the potential to provide the necessary momentum to improve the delivery of services. Furthermore, providing performance-based incentives for product-oriented services is associated with improved delivery of those services and may also have important spillover effects on information-oriented services. Given the continued debate on the appropriate incentives for FLW motivation and performance, further research is needed to identify appropriate combinations of incentives tailored to the context of programs.

## Notes

**Peer Reviewed**
